# Optimal homeostatic stress to maximize the homogeneity of adaptations to interval interventions in soccer players

**DOI:** 10.3389/fphys.2024.1377552

**Published:** 2024-04-09

**Authors:** Mohsen Sheykhlouvand, Mohammadali Gharaat

**Affiliations:** ^1^ Department of Exercise Physiology, Faculty of Sport Sciences, University of Guilan, Rasht, Iran; ^2^ Department of Physical Education, Farhangian University, Tehran, Iran

**Keywords:** cardiorespiratory fitness, bio-motor abilities, athletic performance, team sport, anaerobic power

## Abstract

This study examined the uniformity of adaptations in cardiorespiratory fitness and bio-motor abilities by analyzing individual responses to measures representing the mentioned qualities. Twenty-four male well-trained soccer players (Age = 26 ± 4 years; stature = 181 ± 3.8; Weight = 84 ± 6.1) were randomized to two groups performing short sprint interval training [sSIT (3 sets of 10 × 4 s *all-out* sprints with 20 s of recovery between efforts and 3 min of rest intervals between sets)] or a time-matched small-sided game [SSG (3 sets of 3 v 3 efforts in a 20 × 15 m area with 3 min of relief in-between)]. Before and after the 6-week training period, aerobic fitness indices, cardiac hemodynamics, and anaerobic power were assessed through a graded exercise test utilizing a gas collection system, noninvasive impedance cardiography, and a lower-body Wingate test, respectively. Also, sport-specific bio-motor abilities were determined by measuring linear speed, change of direction, and jumping ability. Comparing inter-individual variability in the adaptive changes by analyzing residuals in individual adaptations indicated that sSIT induces more uniform changes in the first and second ventilatory threshold (VT_1_ & VT_2_), stroke volume, and peak power output across team members than SSG. SSG also yielded lower proportions of responders in $\dot{V}O_{2\max}$ , VT_1_, VT_2_, peak, and average power output compared to sSIT. Additionally, the coefficient of variation in mean group changes in measures of aerobic fitness and bio-motor abilities in response to sSIT were lower than in SSG. Short sprint interval training induces more homogenized adaptations in measures of cardiorespiratory fitness and anaerobic power than small-sided games across team members.

## 1 Introduction

Typically, responses to different exercise interventions are presented as average group values, with the presumption that these values represent individual responses. However, individual adaptations to a standardized intervention vary among athletes with different physiological profiles and locomotor abilities (Mann et al., 2014). Moreover, the quantity of workload imposed by some sport-specific interventions could be unequal due to the inability of intervention to control several influencing factors in workload (Hill-Haas et al., 2008a, 2008b). Such non-uniform physical stresses may result in heterogeneous adaptations among athletes of a group or members of team sports (Clemente et al., 2020; Sandford et al., 2021).

Soccer is a famous team sport characterized by intermittent explosive movements (i.e., acceleration and deceleration, repetitive short-distance sprinting, jumping, and turning in different directions) fueled by the anaerobic metabolic pathway (Rampinini et al., 2011; Nyberg et al., 2016; Mohr et al., 2023). Also, heightened aerobic capacity prevails during less intense activities to accelerate recovery and contribute to sustaining efforts throughout matches. Hence, it is essential to focus on improving both anaerobic and aerobic metabolic pathways to enhance soccer players' performance during a game (Nyberg et al., 2016; Arazi et al., 2017). Various methods have been developed to improve these attributes. High-intensity interval training (HIIT), in its various forms, has proven to be an effective intervention for boosting both cardiorespiratory fitness and anaerobic power in soccer athletes (Dai and Xie, 2023). Similar to the nature of HIIT, soccer involves repetitive, intense actions interspersed with rest intervals of low to moderate intensity (Laursen and Buchheit, 2019). Hence, HIIT could be considered a sport-specific approach to enhance the metabolic conditioning of soccer players.

Several HIIT prescribing methods have been proposed for soccer players, aiming to ensure athletes achieve the necessary exercise intensity during their training sessions in a controlled and sport-specific manner (Laursen and Buchheit, 2019). According to the important principle of training specificity and considering the technical and tactical demands associated with team sports, HIIT in the form of Small-Sided Games [SSGs (i.e., so-called game-based conditioning)] have received exponential growth in interest (Clemente et al., 2021) since the publication of the first systematic review on the topic (Hill-Haas et al., 2011). This is because SSGs simulate the dynamics of an official game while allowing for specific adjustments to emphasize particular behaviors and actions (Clemente, 2020). Given the potency of enhancing physical performance, technical skills, and tactical awareness concurrently, SSGs are considered a time-efficient training approach (Arcos et al., 2015). Nevertheless, despite the mentioned training effectiveness, SSGs have their limitations. Variances in the quantity of strenuous activities performed during SSGs result in inter-individual differences in workload. This results in heterogeneous adaptations to the training among team members (Hill-Haas et al., 2008a; 2008b, and 2011). These constraints support utilizing a less specific yet more controlled HIIT format, such as running-based repeated sprints (Laursen and Buchheit, 2019). As these interventions are performed at maximum effort, they can be prescribed without assessing an individual’s physiological limits (e.g., maximum oxygen uptake [ $\dot{V}O_{2\max}$ ]) to adjust the exercise intensity (Laursen and Buchheit, 2019). However, due to the considerable psychological demands and the extreme physical exertion, it is speculated that athletes with different levels of physical fitness represent varied tolerance to traditional sprint interval training, which is performed as 30–45 s *all-out* sprints. This variability can induce diverse mechanical stress, unidentical physiological demands, and non-uniform adaptations among team members (Sandford et al., 2021).

Recent research has suggested that utilizing shorter-duration sprints, known as short sprint interval training (sSIT) with efforts lasting between 3 and 10 s, could be considered an alternative to the traditional repeated sprint training. This approach has the potential to generate similar adaptive responses while simultaneously improving enjoyment and reducing the perceived exertion rate (Flores et al., 2018; McKie et al., 2018). Implementing sSIT has been proven to generate elevated mechanical responses and decreased peripheral fatigue. This outcome is attributed to the dependence on the ATP-PCr pathway and a reduction in glycolytic activity (Boullosa et al., 2022). Given that the majority of sprints in a soccer match are shorter than 10 m and involve a combination of explosive (rapid acceleration) and leading (gradual acceleration) movements (Dupont and McCall, 2016), sSIT might be considered a sport-specific intervention for soccer players. Nevertheless, limited studies regarding the effects of sSIT on sport-specific performance measures restrict capturing a complete picture of its potency in improving cardiorespiratory fitness and bio-motor abilities in soccer players. For coaches to effectively design programs to improve players’ proficiency in soccer games, they must thoroughly understand the varied responses to different types of HIIT interventions. Accordingly, this study aimed to compare the magnitude of the adaptations to SSGs and sSIT and inter-individual variability in adaptive responses in well-trained soccer players. Given that the workload of sSIT is more controllable than SSGs, we hypothesized that sSIT would result in more homogenized adaptations among team members than SSG.

## 2 Materials and methods

### 2.1 Study design

**FIGURE 1:**
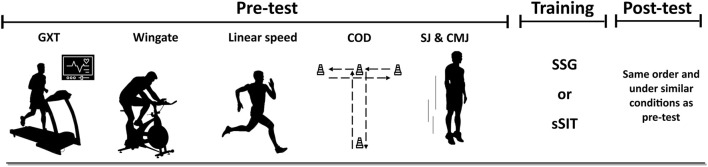
Overview of the experimental protocol. GXT, graded exercise test; COD, change of direction; SJ, squat jump, CMJ, countermovement jump; SSG, small-sided game; sSIT, short sprint interval training.

Figure 1 represents an overview of the experimental protocol. The research utilized a randomized controlled trial featuring two experimental groups. Before the commencement of the study, participants underwent a set of assessments, including both lab- and field-based measurements, to assess cardiorespiratory fitness indicators and soccer-specific bio-motor abilities. Participants underwent a graded exercise test on the initial visit to assess maximum oxygen uptake ( $\dot{V}O_{2\max}$ ) and associated physiological variables. Subsequently, the second visit involved the measurement of Wingate-based anaerobic power. Jumping ability was measured on the third testing session, and change of direction (COD) and linear speed were evaluated on the fourth occasion. Testing sessions were separated by a 24-h recovery period, during which participants were instructed to abstain from consuming caffeine and alcohol, as well as to avoid engaging in strenuous exercise (Gharaat et al., 2020; Barzegar et al., 2021). Approximately 48 h following the last testing session, participants commenced the 6-week training program, and 48 h after the final training session, they underwent the identical testing procedure, following the same order and under the same conditions as the pre-test. Measurements were carried out by two expert technicians who were blinded to the interventions.

### 2.2 Participants

Twenty-four male well-trained soccer players [two goal keepers, six right and left fullbacks, four center backs, eight midfielders (center and wing), and four forwards] provided written informed consent and voluntarily participated. Participants were members of a provincial-level club with a soccer-playing experience of at least 10 years. Following the medical screening for any unknown complication or physical injury putting the participants at risk of high-intensity exercise, players were matched based on playing position and were randomized to either sSIT (Age = 25.8 ± 3.5 years; stature = 182 ± 2.9; Weight = 83.1 ± 4.8) or SSG (Age = 26.2 ± 4 years; stature = 180 ± 3.1; Weight = 84.9 ± 5.3) groups, each of 12. All players were familiar with all-out interval interventions but had not engaged in sSIT or SSG over the 3 months preceding this study. All procedures adhered to the ethical standards outlined in the Helsinki Declaration and were approved by the ethical committee of the University of Guilan, Iran.

### 2.3 Graded exercise test

After 10 min of warm-up consisting of 5 min of jogging and 5 min of dynamic stretching, athletes completed the graded exercise test on a treadmill (NordicTrack 1750, United States). The test commenced with the initial intensity of 8 km·h^−1^, and velocity incrementally increased by 1 km h^−1^ every 3 min. Stages were separated with 30 s of relief intervals during which the blood lactate concentration was measured by earlobe blood sampling. During the test, a breath-by-breath gas collection system (MetaLyzer 3B-R2, Cortex, Germany) continuously measured cardiorespiratory fitness measures. $\dot{V}O_{2\max}$ was verified if at least three of the following criteria were met: a) plateau or a slight drop in $\dot{V}O_{2}$ despite increasing workload; B) respiratory exchange ratio (RER) exceeding 1.1; C) attaining ≥90% age-predicted heart rate; D blood lactate concentration reaching 8 mmol l^−1^; E) visible exhaustion (Sheykhlouvand et al., 2015; Sheykhlouvand and Forbes, 2017; Dai and Xie, 2023). VT_1_ was defined as the point at which there was a raise in ${\dot{V}}_{E}/\dot{V}O_{2}$ and end-tidal O_2_ tension (P_ET_O_2_) occurred without a simultaneous elevation in ${\dot{V}}_{E}/\dot{V}{\text{CO}}_{2}$ . The criterion for identifying VT_2_ was the sustained increase in both the ${\dot{V}}_{E}/\dot{V}O_{2}$ and ${\dot{V}}_{E}/\dot{V}{\text{CO}}_{2}$ ratio curves, correlated with the decrease in P_ET_O_2_ (Fereshtian et al., 2017; Alejo et al., 2022; Dai and Xie, 2023). Also, cardiac output (CO) and stroke volume (SV) were non-invasively measured using impedance cardiography (PhysioFlow, Manatec, France). Electrodes of the device were placed on the neck (two electrodes), xiphoid sternum (two electrodes), and one on each side of the chest. Following 20 s of calibration, cardiac hemodynamics were continuously measured during the test (Sheykhlouvand et al., 2022).

### 2.4 Lower-body Wingate test

Participants underwent a 30-s all-out Wingate test to determine their peak power output (PPO) and average power output (APO). At the start of the test, participants were directed to pedal against the inertial resistance of the ergometer (894E, Monark, Sweden) at their maximum speed. Following that, a resistance corresponding to 0.075 kg/kg of body mass was imposed, and the electronic revolution counter was initiated. Continuous verbal encouragement was given during the test, and PPO and APO were computed using the device’s software.

### 2.5 Jumping ability

To assess jumping ability, tests for vertical jump (VJ) and countermovement jump (CMJ) were conducted utilizing a Globus electronic contact mat system (Codognè, Italy). The maximum height reached was measured with a precision of 0.01 m. During VJ, participants positioned their hands on their hips, shoulders, and feet wide apart and flexed their knees to approximately a 90-degree angle for 3 s. Subsequently, they exerted maximum effort in executing a vertical jump (Ramirez-Campillo et al., 2013). For executing CMJ, participants were guided to place their hands on their hips, assume a stance with feet and shoulders spread widely apart, and execute a downward motion (without any constraints on the achieved knee angle) before engaging in a vertical jump with full effort (Ojeda-Aravena et al., 2021). Participants were directed to land in an upright position and bend their knees after landing. Each participant performed three trials of this task, with rest intervals of approximately 60 s between each trial. The trial yielding the best performance among the three attempts was chosen for further statistical analysis.

### 2.6 Linear speed

Following a comprehensive warm-up, participants underwent two consecutive 20-m sprint tests with a 3-min rest interval to assess their linear speed. Participants were prompted to sprint between electronic timing gates (JBL Systems Oslo, Norway) at their maximum speed, choosing their own start time, with the time measured to the nearest 0.01 s. Upon readiness, the players commenced the run, starting from a stationary position behind the starting line. The trial exhibiting the highest performance level was selected for further statistical analysis.

### 2.7 Change of direction

The evaluation of Change of Direction (COD) performance involved measuring multi-directional running speed, including linear sprints, shuffling to the right and left, and backpedaling, utilizing the MAT-test. This test is a modified version of the agility T-test. Due to the repeated sprints with direction changes over a short distance, MAT appears to be a more reliable and sport-specific measure of agility compared to the T-test (Sassi et al., 2009).

### 2.8 SSG and sSIT protocols

The players followed their regular soccer off-season training schedule, engaging in tactical drills, technical exercises, and simulated competitive games, five sessions per week. These sessions typically lasted between 60 and 70 min, from 4:00 to 6:00 P.M. sSIT and SSG were completed before the regular soccer training program on Sunday, Monday, and Wednesday. The training was initiated with ∼5 min of jogging, 5–10 min of dynamic movements, and sprinting with the integration of soccer-specific technical actions. Following the warm-up, participants of the sSIT group executed 3 sets of 10 × 4 s all-out sprints with 20 s of recovery between efforts and 3 min of rest intervals between sets. The SSG comprised 3 sets of time-matched (4 min each) 3 v 3 efforts in a 20 × 15 m area with 3 min of relief between efforts. SSG was executed without a goalkeeper, while two coaches encouraged players and supplied new balls to maintain the game’s flow. Teams were balanced according to the participants’ playing positions. The game’s objective was to maintain possession of the ball as long as possible (Hill-Haas et al., 2011; Dellal et al., 2012)

### 2.9 Statistical analysis

Data are presented as mean ± SD. The normality of distribution was assessed through the Shapiro-Wilk test, while Levene’s test was employed to evaluate the homogeneity of variances. A two-factor mixed analysis of variance with the between factor [group (SSG & sSIT)] and repeated factor [trial (pre-training & post-training)] analyzed the data. Main effects or significant interactions were subsequently analyzed using Tukey’s post-hoc. Inter-individual variability in the adaptive changes was measured using three methods. First, individual percent changes from pre-to post-training were calculated for each variable, and the coefficient of variations (CVs) in the adaptive changes was determined as the ratio of SD to mean individual percent changes. Second, individual residuals in percent changes were calculated as the squared root of the squared difference between the individual percent change and the mean percent change for each tested variable, and the group mean residuals for each intervention were compared between SSG and sSIT interventions to determine their effects on inter-subject variability in the magnitude of the adaptations. Third, technical error (TE) was calculated for each variable (TE = SD_*diff*_/ $\sqrt{2}$ ) (Hopkins, 2000) to determine responders (Rs) and non-responders (NRs) to the interventions. In accordance with Hopkins (2000), a change greater than 2 × TE was representative of a high probability (with odds of 12 to 1) that the observed response is an actual physiological adaptation beyond what could reasonably be ascribed to technical or biological fluctuations. NRs were characterized as individuals unable to exhibit a notable increase or decrease (favoring beneficial changes) in the measured variables exceeding 2 × TE from zero (Ramirez-Campillo et al., 2018). TEs were as follows [ $\dot{V}O_{2\max}$ , 0.825 (ml·kg^−1^·min^−1^) × 2; VT_1_, 0.707 (% $\dot{V}O_{2\max}$ ) × 2; VT_2_, 0.771 (% $\dot{V}O_{2\max}$ ) × 2; CO, 0.225 (l min^−1^); SV, 1.311 (ml·b^−1^) × 2; PPO, 12.675 (W) × 2; APO, 9.205 (W) × 2; SJ, 0.575 (cm) × 2; CMJ, 0.463 (cm) × 2; 20-m sprint, 0.020 (s) × 2; and COD, 0.069 (s) × 2]. Finally, The Chi-Square test (χ^2^) was utilized to compare groups of participants falling within the two times the typical error (2 × TE) range calculated for each outcome (NRs) or those exceeding it by more than two times the typical error (Rs). Statistical analyses were carried out using SPSS software, version 25.0 (IBM Corp., Chicago, IL), and the alpha level was set at 0.05.

## 3 Results

At the baseline, no between-group difference was observed for the measured physiological and anthropometric measures. Both SSG and sSIT significantly enhanced bio-motor abilities and cardiorespiratory fitness measures over time (Tables 1, 2). A significant time-regimen interaction was found in linear speed. The change in 20-m sprint time in response to sSIT was significantly greater (*p* = 0.005; 95% CI: 0.030–0.146) than that of SSG.

**TABLE 1:** Change in measures of cardiorespiratory fitness over time.

	SSG			sSIT	
	Pre	Post		Pre	Post
$\dot{V}O_{2\max}$ (ml·kg^−1^·min^−1^)	51.84 ± 2.6	53.87 ± 2.1		51.57 ± 2.6	54.46 ± 2.7
*p-value*	0.001^†^			0.0004^†^	
*%∆*	3.9			5.6	
*d*	0.85			1.07	
*CV of mean %∆*	38%			16%	
${\dot{V}}_{E}$ (l·min^−1^)	159.7 ± 6.9	165.9 ± 7.0		160.6 ± 7.8	169.0 ± 8.1
*p-value*	0.002^†^			0.0006^†^	
*%∆*	3.9			5.7	
*d*	0.89			1.05	
*CV of mean %∆*	35%			17%	
VT_1_ (% $\dot{V}O_{2\max}$ )	71.9 ± 3.1	74.7 ± 3.7		72.9 ± 3.7	76.9 ± 3.9
*p-value*	0.003^†^			0.0001^†^	
*%∆*	3.9			5.4	
*d*	0.82			1.05	
*CV of mean %∆*	48%			14%	
VT_2_ (% $\dot{V}O_{2\max}$ )	87.0 ± 3.2	90.0 ± 2.2		87.7 ± 3.0	91.3 ± 2.3
*p-value*	0.001^†^			0.0003^†^	
*%∆*	3.4			4.1	
*d*	1.09			1.34	
*CV of mean %∆*	61%			29%	
CO (l·min^−1^)	31.1 ± 1.5	32.4 ± 1.4		30.2 ± 1.7	31.8 ± 1.8
*p-value*	0.002^†^			0.0008^†^	
*%∆*	4.2			5.3	
*d*	0.89			0.91	
*CV of mean %∆*	48%			28%	
SV (ml·b^−1^)	156.2 ± 7.2	161.9 ± 6.6		160.5 ± 7.7	167.4 ± 6.6
*p-value*	0.008^†^			0.0001^†^	
*%∆*	3.6			4.3	
*d*	0.82			0.96	
*CV of mean %∆*	57%			32%	

Values are means ± SD; %∆, within group changes from pre-to post-training.

CO, cardiac output; ES, effect size; SV, stroke volume; $\dot{V}O_{2\max}$ maximum oxygen uptake; ${\dot{V}}_{E}$ , maximal ventilation; VT_1_, first ventilatory threshold; VT_2_, second ventilatory threshold. N = 10 for each group. ^†^ Significantly greater than pre-training value (*p* < 0.05).

**TABLE 2:** Change in measures of bio-motor abilities over time.

	SSG		sSIT	
	Pre	Post	Pre	Post
PPO (W)	873.3 ± 60.0	914.3 ± 55.2	889.7 ± 60.5	937.7 ± 59.8
*p-value*	0.007^†^		0.001^†^	
*%∆*	4.7		5.4	
*ES*	0.71		0.81	
*CV of mean %∆*	55%		24%	
APO (W)	541.5 ± 43.7	572.7 ± 45.6	543.8 ± 39.4	583.2 ± 39.3
*p-value*	0.004^†^		0.0004^†^	
*%∆*	5.7		7.2	
*ES*	0.69		1.00	
*CV of mean %∆*	47%		21%	
20-m sprint (s)	3.30 ± 0.04	3.19 ± 0.07	3.23 ± 0.08	3.04 ± 0.07
*p-value*	0.004^†^		0.0007^†^	
*%∆*	−3.4		−6.2⁑	
*ES*	0.92		1.52	
*CV in mean %∆*	53%		28%	
COD (s)	7.90 ± 0.22	7.65 ± 0.20	7.91 ± 0.20	7.67 ± 0.21
*p-value*	0.002^†^		0.001^†^	
*%∆*	−3.2		−3.1	
*ES*	1.19		1.17	
*CV of mean %∆*	30%		28%	
SJ (cm)	38.4 ± 1.5	40.4 ± 1.2	38.6 ± 1.5	40.8 ± 1.6
*p-value*	0.005^†^		0.009^†^	
*%∆*	5.2		5.6	
*ES*	1.47		1.42	
*CV of mean %∆*	32%		29%	
CMJ (cm)	42.0 ± 1.0	44.2 ± 1.1	41.7 ± 1.2	44.0 ± 0.9
*p-value*	0.004^†^		0.002^†^	
*%∆*	5.2		5.5	
*ES*	1.09		1.16	
*CV of mean %∆*	24%		25%	

Values are means ± SD; %∆, within group changes from pre-to post-training.

APO, average power output; COD, change of direction; CMJ, countermovement jump; ES, effect size; PPO, peak power output; SJ, squat jump. N = 10 for each group. ^†^ Significantly greater than pre-training value (*p* < 0.05); ** Significantly greater changes than the SSG (*p* < 0.05).

**FIGURE 2:**
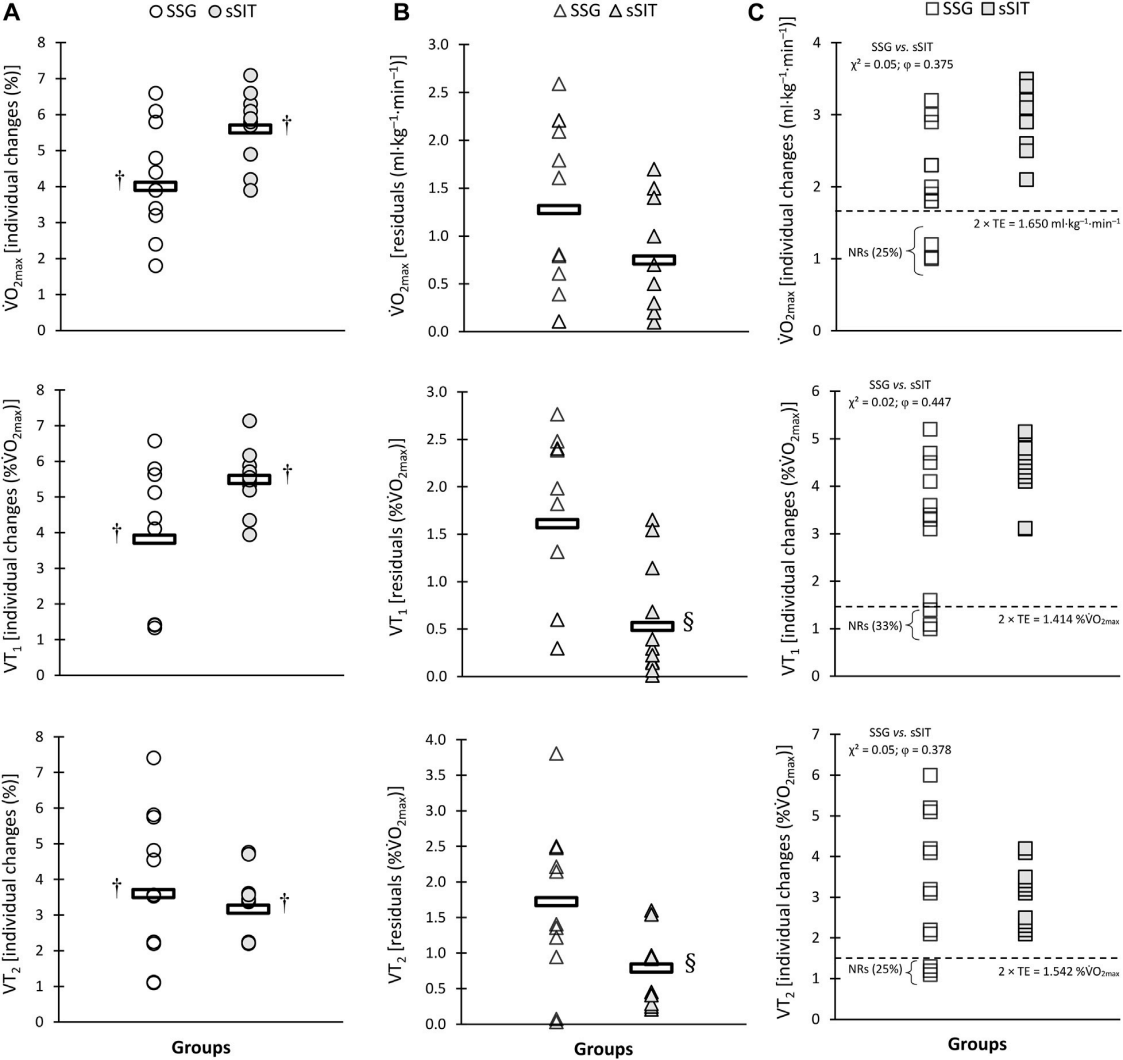
Individual percent change **(A)**, residuals in individual changes **(B)**, and proportions of non-responders **(C)** to small-sided game (SSG) and short sprint interval training (sSIT) over the training period. $\dot{V}O_{2\max}$ , maximum oxygen uptake; VT_1_, first ventilatory threshold; VT_2_, second ventilatory threshold. ^†^ Significantly greater than pre-training value. ^§^ Significantly lower than SSG group.

**FIGURE 3:**
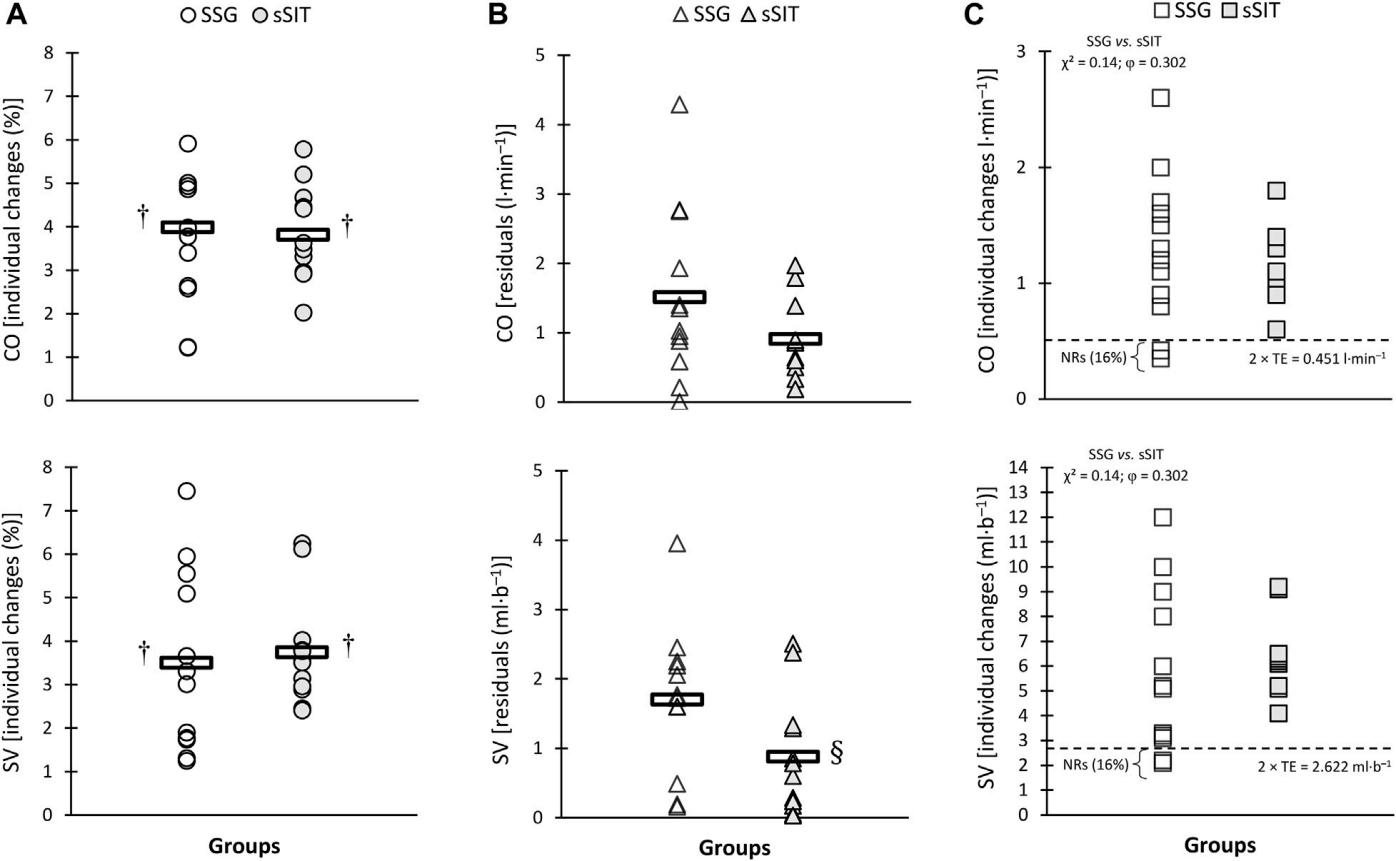
Individual percent change **(A)**, residuals in individual changes **(B)**, and proportions of non-responders **(C)** to small-sided game (SSG) and short sprint interval training (sSIT) over the training period. CO, cardiac output; SV, stroke volume. ^†^ Significantly greater than pre-training value. ^§^ Significantly lower than SSG group.

**FIGURE 4:**
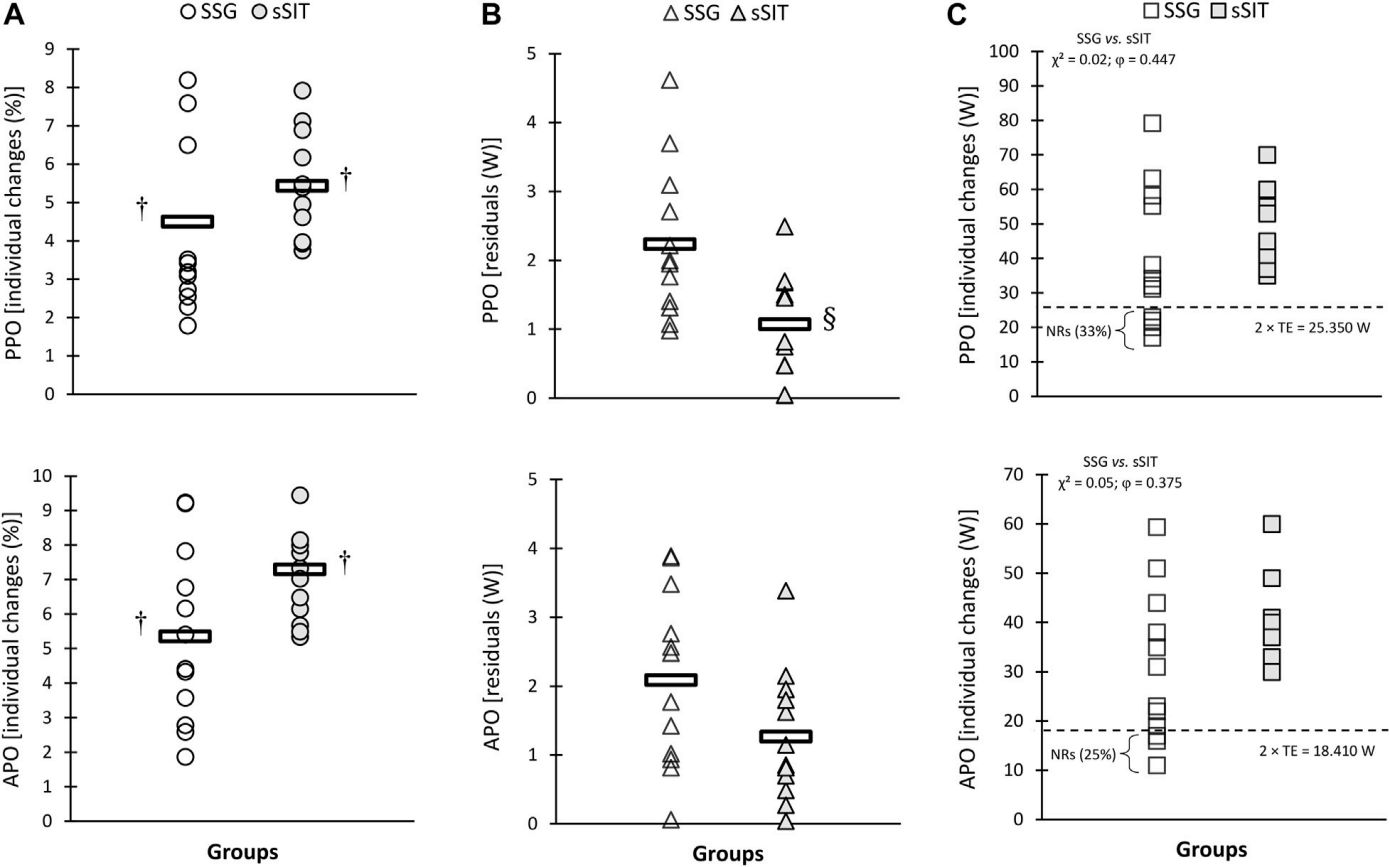
Individual percent change **(A)**, residuals in individual changes **(B)**, and proportions of non-responders **(C)** to small-sided game (SSG) and short sprint interval training (sSIT) over the training period. PPO, peak power output; APO, average power output. ^†^ Significantly greater than pre-training value. ^§^ Significantly lower than SSG group.

**FIGURE 5:**
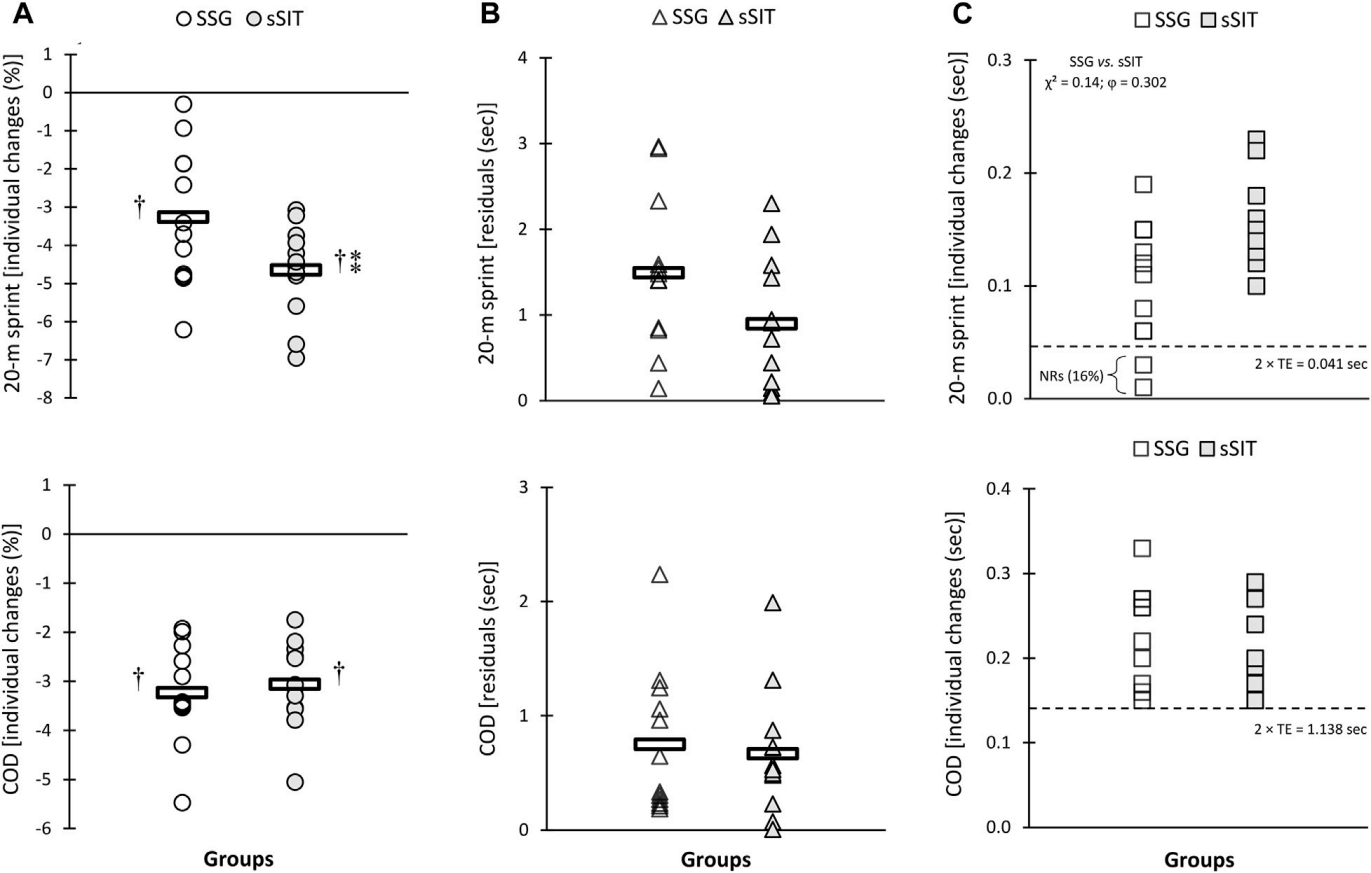
Individual percent change **(A)**, residuals in individual changes **(B)**, and proportions of non-responders **(C)** to small-sided game (SSG) and short sprint interval training (sSIT) over the training period. COD, change of direction. ^†^ Significantly greater than pre-training value. ** Significantly greater than SSG group.

**FIGURE 6:**
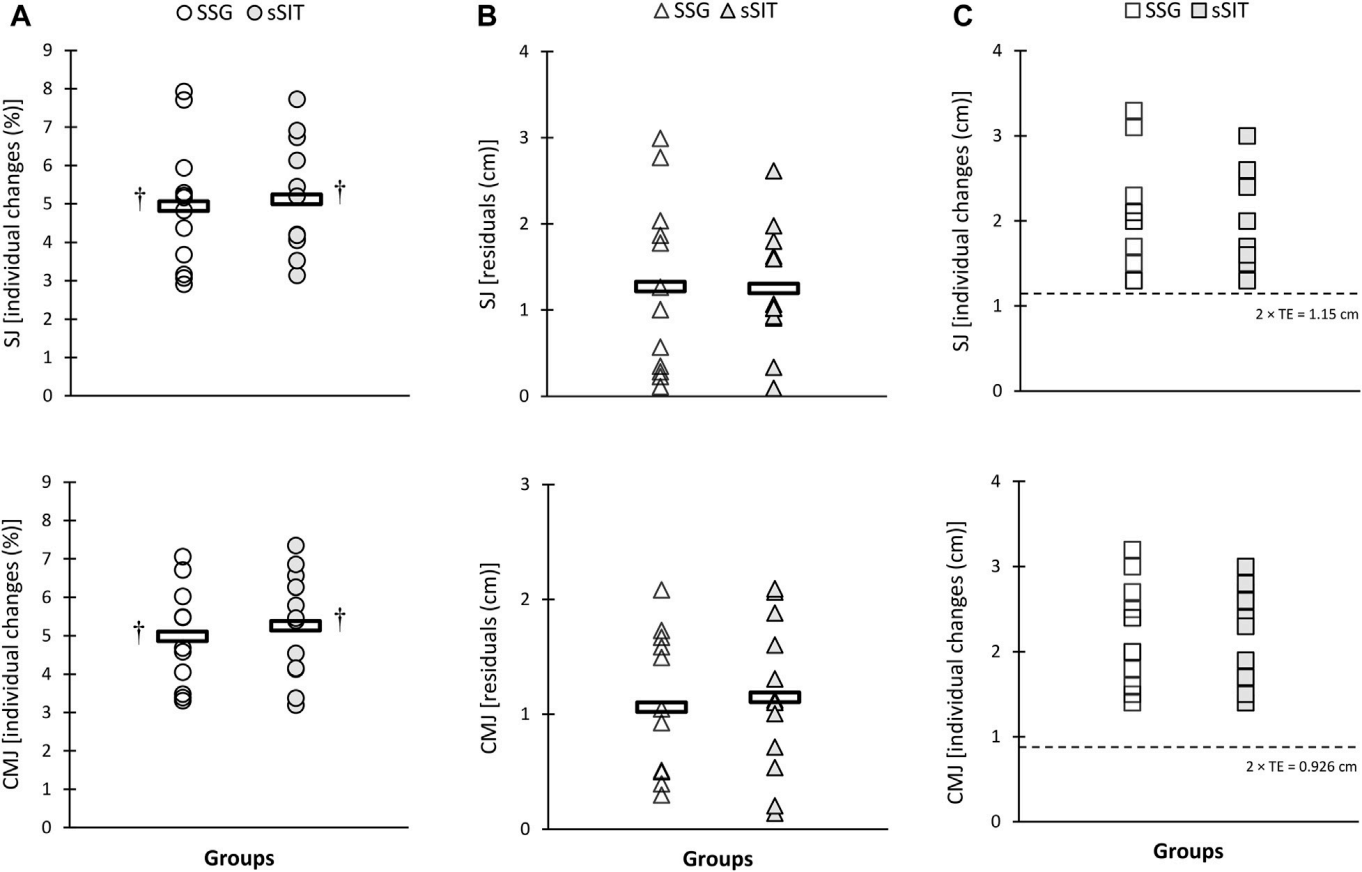
Individual percent change **(A)**, residuals in individual changes **(B)**, and proportions of non-responders **(C)** to small-sided game (SSG) and short sprint interval training (sSIT) over the training period. SJ, squat jump; CMJ, countermovement jump. ^†^ Significantly greater than pre-training value.

After the 6-week training period, a significant time-regimen interaction (*p* ≤ 0.05) was found in residuals of individual changes in VT_1_, VT_2_, SV, PPO, and APO. As shown in Figures 2–4, sSIT resulted in lower residuals in individual changes in VT_1_ (*p* = 0.003; 95% CI: 0.419–1.752), VT_2_ (*p* = 0.012; 95% CI: 0.245–1.798), SV (*p* = 0.048; 95% CI: 0.008–1.642), and PPO (*p* = 0.007; 95% CI: 0.357–1.966) compared to SSG. Moreover, except for SJ and CMJ, the coefficient of variations in mean percent changes in response to sSIT were lower than those of SSG (Tables 1, 2). Moreover, SSG showed 25%, 33%, 25%, 16%, 16%, 33%, and 25% non-responders in $\dot{V}O_{2\max}$ , VT_1_, VT_2_, CO, SV, PPO, and APO, respectively (Figures 2–4). χ^2^ test indicated lower responders to SSG in $\dot{V}O_{2\max}$ (*p* = 0.05, φ = 0.375), VT_1_ (*p* = 0.02, φ = 0.447), VT_2_ (*p* = 0.05, φ = 0.378), PPO (*p* = 0.02, φ = 0.447), and APO (*p* = 0.05, φ = 0.378) than sSIT (Figures 2, 4). No between-group difference was found in the residuals of the adaptations and proportions of responders in linear speed, change of direction, and jumping ability (Figures 5, 6).

## 4 Discussion

This study compared the homogeneity of the adaptations to small-sided games versus short sprint interval training by evaluating inter-individual variability in adaptive responses of cardiorespiratory fitness and bio-motor abilities in soccer players. Both SSG and sSIT interventions adequately stimulated the adaptive mechanisms, enhancing qualities mentioned above. The most remarkable findings of this study were that, when comparing inter-individual variability in terms of residuals in the magnitude of the changes over the 6-week training period, sSIT resulted in more uniform adaptations in VT_1_, VT_2_, SV, and PPO than SSG. From a “responders and non-responders” point of view, SSG indicated significantly greater proportions of non-responders in $\dot{V}O_{2\max}$ , VT_1_, VT_2_, PPO, and APO than sSIT. In addition, except for jumping ability, the coefficient of variations in mean group changes in response to sSIT was lower than those of SSG.

Our study stands as the first endeavor to investigate how adaptations manifest in response to the small-sided game and sprint interval training multi-dimensionally and to provide a clear picture of individual adaptive responses to external stimuli imposed by these interventions. Our results corroborate previous studies indicating remarkable heterogeneity in individual adaptations to different forms of interval interventions (Dai and Xie, 2023; Wang and Zhao, 2023; Luo et al., 2024). Divergence in the adaptations around mean alludes to inter-subject variation in the adaptive response to training intervention, a phenomenon frequently observed yet explicitly addressed in only a relatively small number of studies. At both ends of a spectrum representing individual responses, there are individuals who exhibit notably significant responses (high Rs) and those who demonstrate notably small responses (low Rs) to a given training intervention (Mann et al., 2014). Nevertheless, individuals exhibiting a limited response to an exercise intervention in one parameter (e.g., $\dot{V}O_{2\max}$ ) may not necessarily demonstrate the same response in other parameters (Vollaard et al., 2009; Scharhag-Rosenberger et al., 2012). This complexity further complicates the concept of high responders versus low responders. Previous studies attribute heterogeneous adaptations to divergent physiological profiles, genotype, baseline phenotype, training status, nutritional strategies, sleep and stress, and fixed vs. flexible training prescription (Mann et al., 2014).

When comparing SSG and sSIT, it is essential to consider the homeostatic stress imposed by the training sessions, in addition to the mentioned parameters above. A game is a dynamic system formed by the interplay between two teams amid diverse contextual factors (Gréhaigne et al., 1997). Accordingly, all training scenarios encompass a certain degree of unpredictability, and this inherent unpredictability naturally contributes to an increase in the variability of stimuli (Hill-Haas et al., 2008a). A resulting source of variability in physiological demands may lead to different degrees of adaptations (Clemente et al., 2020). The collective homeostatic stress during an exercise session is influenced by factors like exercise intensity and duration, acting as a “stimulus” that triggers adaptive responses (Mann et al., 2014). “At the cellular level, adaptation to exercise training results from the cumulative impact of specific transcriptional and translational “micro-adaptations” occurring after each exercise session (Flück, 2006). Consequently, differences in the acute exercise stimulus received may account for individual variations in the training responses accumulated over time (Mann et al., 2014).” To modify such heterogeneity, researchers employ different reference intensities such as proportions of $\dot{V}O_{2\max}$ , MHR, anaerobic speed reserve, and different durations tailored to individual exercise tolerance or characteristics of the game in which the athlete competes. While these methods generate almost similar homeostatic stress levels across individuals with varying physiological capacities, they do not necessarily standardize all aspects of influencing parameters (McPhee et al., 2010). Research indicates that at higher intensities, lower variability in measured physiological parameters occurs (Bagger et al., 2003), and by exercising at higher absolute intensities, participants are likely to control their exercise intensity within a closer bandwidth (O’Grady, 2021). Accordingly, we utilized the upper limit of supramaximal intensity (*all-out*) for performing sSIT to fully engage individuals’ capacity. By doing so, all factors influencing the utilization of the proportions of an individual’s physiological and locomotor capacities are considered. As a result, athletes underwent the same proportion of their capacity (100%) and encountered equivalent homeostatic stress. However, SSG failed to modify the abovementioned parameters, resulting in a considerable variation in the adaptive responses.

Another important finding of this study was the significant effects of sSIT and SSG on enhancements of cardiorespiratory fitness and bio-motor abilities. Enhancements in cardiorespiratory fitness could be attributed to either an increase in the oxygen delivery (i.e., central component) or the improved ability of the active muscles to utilize delivered oxygen (i.e., peripheral component) (Sheykhlouvand et al., 2016a; Sheykhlouvand et al., 2016b; Sheykhlouvand et al., 2018a; Rasouli Mojez et al., 2021; Sayevand et al., 2022).

The rise in stroke volume and cardiac output following sSIT and SSG could partially contribute to improving of the central component of cardiorespiratory fitness. Our findings additionally validate the efficacy of both interval interventions in enhancing Wingate-based anaerobic power and jumping ability, the latter indicative of muscular power. Potential explanations for the enhanced anaerobic power may involve an increased discharge rate and recruitment of high-threshold motor units (Dolci et al., 2020), elevated total creatine content in active muscles (Hoffmann et al., 2020), and an improved buffering capacity of muscles (Sheykhlouvand et al., 2018b). Linear speed and change of direction also significantly improved in both groups. Notably, the magnitude of changes in 20-m sprint time in response to sSIT was significantly greater than in SSG, possibly due to the repeated generation of sprints during sSIT. A quick COD may arise from rapid force development and high power generation by the lower extremities (Miller et al., 2006). The improved linear speed can be attributed to enhancements in the acceleration component of maximal sprinting and improvements in stride length, contributing to overall sprint gains (Lee et al., 2020).

One potential limitation of this study is the exclusive inclusion of male participants, which restricts the generalizability of the findings solely to males, not females. Additionally, our capacity to closely supervise participants’ sleep quality and rigorously monitor dietary habits was limited. It is essential to highlight that our results are related explicitly to interval protocols conducted under the conditions of this study. The potential for similar outcomes with other reference intensities or training volumes remains unknown.

## 5 Conclusion

In conclusion, this study compared the homogeneity of bio-motor abilities and cardiorespiratory fitness adaptations to small-sided games and short sprint interval training. Both recruited protocols adequately stimulated the mechanisms responsible for enhancing measures representing the abovementioned qualities. Comparing inter-individual variability in the adaptive changes by analyzing residuals in individual adaptations indicated that sSIT induces more uniform changes in VT_1_, VT_2_, SV, and PPO across team members than SSG. SSG also yielded lower responders in $\dot{V}O_{2\max}$ , VT_1_, VT_2_, PPO, and APO compared to sSIT. Additionally, excluding jumping ability, the coefficient of variation in mean group changes in response to sSIT was lower than in SSG.

## 6 Practical applications

Our findings indicated that 6 weeks of short sprint interval training facilitates more homogenous adaptations in measures of cardiorespiratory fitness and anaerobic power compared to a time-matched small-sided game. Imposing a uniform external load to elicit the same degrees of adaptive responses across team members is one of the crucial objectives of preparatory programs. Although SSG considers the game’s technical and tactical aspects, it fails to impose external load on team members consistently. Hence, when the objective of the intervention is to uniformly enhance aerobic and anaerobic qualities across team members, it is recommended that sSIT be incorporated into regular soccer training under the conditions of this study.

## Data Availability

The raw data supporting the conclusion of this article will be made available by the authors, without undue reservation.
